# The foraging ecology of yellow-billed and red- billed choughs changed between two climatically different years

**DOI:** 10.1038/s41598-023-46336-0

**Published:** 2023-11-27

**Authors:** Antonio Rolando, Cecilia Basso, Nicolò Brunelli, Massimo Bocca, Alex Laini

**Affiliations:** 1 Department of Life Sciences and Systems Biology, Turin University, via Accademia Albertina 13, 10123 Turin, Italy; 2 Société de la Flore Valdôtaine, via J. B. de Tillier 3, 11100 Aosta, Italy

**Keywords:** Ecology, Behavioural ecology, Biodiversity

## Abstract

Climate change is affecting the alpine ecosystem at an unprecedented rate, with marked changes in spring phenology and the elevation distribution of birds. Changes in the European Alps are happening rapidly, and it is possible behaviours stand to change from one year to the next. The year 2022 was characterised by climatic extremes: Italy experienced its hottest year ever, and it was the driest since 1800. Here, we assessed whether the foraging ecology of two coexisting upland bird species, the yellow-billed and the red-billed chough, changed from 2021 to 2022. We assessed foraging stay times, flock size, propensity to mixed flocking, foraging home ranges and altitudinal distribution. Stay times of both species when foraging in monospecific flocks significantly shortened in 2022, especially in the case of the red-billed chough. The two corvids are known to influence each other when foraging together. In 2021, as expected, the stay times of the red-billed chough decreased when in the presence of the congener, but this did not occur in 2022. Instead, the yellow-billed chough increased its altitudinal foraging distribution in 2022. The results are in line with the hypothesis that large climate variations may disrupt the foraging ecology of mountain birds. However, as it is not possible to draw solid conclusions from just two years of observations, further field research will have to be planned in the future.

## Introduction

Climate change is affecting the upland ecosystems at an unprecedented rate. Since 1970, rising temperatures have induced marked changes in spring phenology and the distribution of animals, plants, and fungi across elevation gradients in the European Alps. Spring phenology has been shifting earlier during the past four decades, and distribution ranges have shown upward trends for most taxonomic groups. However, except for terrestrial insects, the upward shift of organisms seems currently too slow to track the pace of isotherm shifts induced by climate warming^1^. As for upland birds, models suggest that open habitat species may face a severe reduction in distribution as grasslands are colonised by encroaching forest and shrubland. This loss may be exacerbated if upward shifts are constrained, either due to a lack of higher altitude areas or to a habitat ‘squeeze’ caused by an asymmetric response of vegetation zones to climate change at higher elevations^2–4^. Such is the case predicted for the rock ptarmigan *Lagopus muta* in the Italian Alps^5^. Due to the loss of suitable habitat, warming will also reduce occupancy ranges of typical alpine bird specialists such as the yellow-billed chough *Pyrrhocorx graculus,* the alpine white-winged snowfinch *Montifringilla nivalis* and the alpine accentor *Prunella collaris*^6^. Distribution changes of upland birds are usually considered to be indirect effects of local warming (i.e., due to vegetation changes), but direct effects of temperature (rising above or dropping below the species’ thermal optimum) have also been identified^7^.

As for spring phenology, earlier breeding seasons are expected to become more frequent in the future. However, migratory species may face greater difficulties than sedentary ones. For example, recent research found that despite the timely arrival of the wheatear *Oenanthe oenanthe* at its breeding sites, the birds were not able to carry out their reproduction activities in synchrony with the environmental conditions^8^. Research in a subalpine habitat also showed that resident bird species track inter-annual variation in spring phenology better than long-distance migrants^9^. In addition to phenological and distributional changes, studies have shown that high mountain bird species are also adapting morphologically to climate change^10^. For instance, Delgado et al. 2019^11^, using a 100-year-long time series, showed that the tarsus length of different snowfinch species (genera *Montifringilla*, *Pyrgilauda* and *Onychostruthus*) has decreased and the saturation of the melanin-based colour has increased, which was correlated with increases in temperature and precipitations. It is also plausible that the body size of upland bird species is declining in response to global warming, in accordance with Bergman’s rule, as recently demonstrated for many other bird species^12–17^. Changes in distribution, phenology and phenotype are happening rapidly—over just a few years to a few decades at most. It follows that certain flexible behaviours, which are dependent on either climatic or climate-controlled factors, may undergo change even more rapidly due to abrupt changes that occur from year to year. Behavioural flexibility has been suggested to be a mechanism for coping with climate change^18,19^. Foraging behaviours could be one of the earliest to switch since it depends on trophic availability, which is modulated by weather conditions.

We studied the foraging ecology of the yellow-billed chough (*Pyrrhocorax graculus*) and the red-billed chough (*Pyrrhocorax pyrrhocorax*) in two consecutive years, namely 2021 and 2022. These years differed significantly in terms of their climate. Data from the European Union's Copernicus Climate Change Service revealed 2022 to be a year of climatic extremes: for Europe, it was the second warmest year and the hottest summer on record^20^, and it had the worst drought for the last 500 years^21^; moreover, Italy experienced its warmest year ever and its driest year since 1800^22^. The yellow-billed and the red-billed chough have a broad, albeit discontinuous, Palearctic distribution, ranging from Europe and north Africa to Central Asia and China. Both species breed on mountains, but the red-billed chough also nests on coastal cliffs. The yellow-billed chough is widespread in the European Alps (it is also known as the Alpine chough), where it has been extensively studied since the 1990s. Research on these breeds has focused on their breeding biology^23^, foraging behaviours^24–26^, local movements^27–29^, survival^30^ and relationships with man^31–34^. In the western Italian Alps, the two species are sympatric and may also be present in the same habitat at the same time (syntopy). In such a condition, interspecific coexistence is enabled through reproductive and dietary segregation. The yellow-billed chough nests in a greater variety of sites and one month later than its congener^29^. The yellow-billed chough is a surface feeder that is mainly frugivorous from September to February (berries), herbivorous-omnivorous in March and April, and insectivorous from May to August (grasshoppers from July onward). Conversely, the red-billed chough is an undersurface feeder (a digger and prober) that can be regarded as prevalently insectivorous-herbivorous, given that it is specialised to catch soil-dwelling insects and to collect bulbs^35,36^. The two species’ foraging strategies are also different. The yellow-billed chough stays for a relatively short time at a feeding patch and feeds quickly. By contrast, the red-billed chough tends to stay at a feeding site for twice as long as the yellow-billed chough, and its feeding time is four times slower^37^. Both species gather in flocks when foraging. The yellow-billed choughs stay for a shorter time in a patch and feed more quickly when in larger flocks. Conversely, flock size does not significantly affect the foraging behaviour of the red-billed chough, probably because the typical flock size tends to remain small. The propensity for mixed-species flocking is rather low. However, when together, the stay times of the red-billed choughs shorten significantly, and the feeding rates of the yellow-billed chough decrease significantly^38^. The main purpose of this study was to assess whether the foraging ecology of yellow-billed and red-billed choughs of an Italian alpine valley significantly changed from 2021 to 2022 as a possible consequence of the climate extremes experienced in 2022.

## Results

### Climate

**Figure 1:**
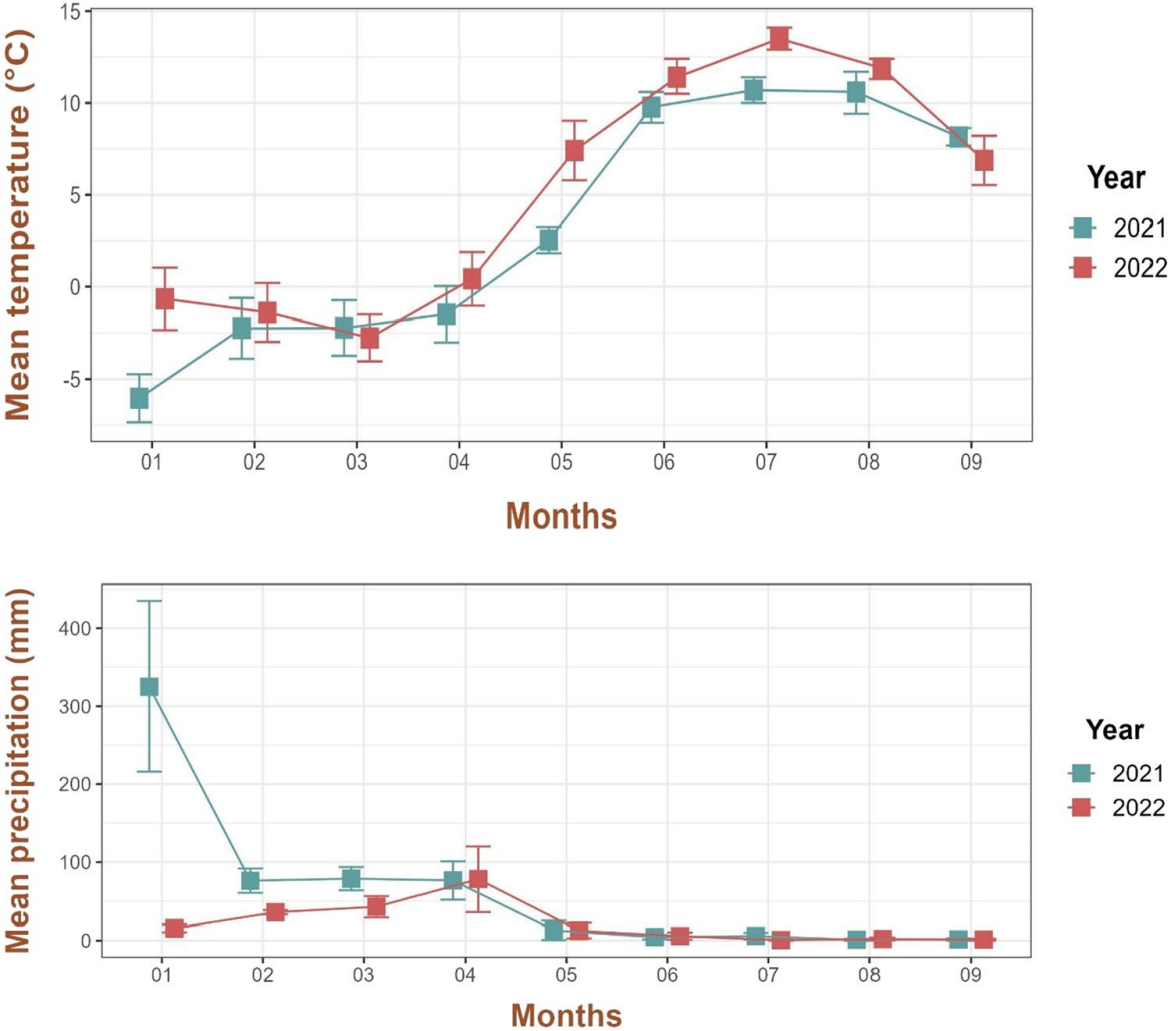
Temperature and precipitation trends in the Dondena basin (Champorcher Valley, Italy) for the months January through to September in the years 2021 and 2022.

In the study area, the months prior to (January to May) and during our study period (June to September) were significantly hotter (Χ^2^_1_ = 26.7, *p* =  < 0.0001) and drier (Χ^2^_1_ = 53.1, *p* =  < 0.0001) (likelihood ratio test) in 2022 than the same months in 2021. However, temperature and precipitation followed different trends. Almost all the months of 2022 were hotter than those of 2021, while only the first three months of 2022 were drier (with less precipitation) than those of 2021 (Fig. 1).

### Stay times

The first overall LMM revealed stay times to vary significantly between species (being shorter in the yellow-billed chough, F_1,667_ = 10.3; *P* < 0.01), year (shorter in 2022, F_1,32_ = 17.0, *P* < 0.001), and flock size (becoming shorter as flock size increased, F_1,633_ = 13.1; *P* < 0.001) (Table S1).

**Figure 2:**
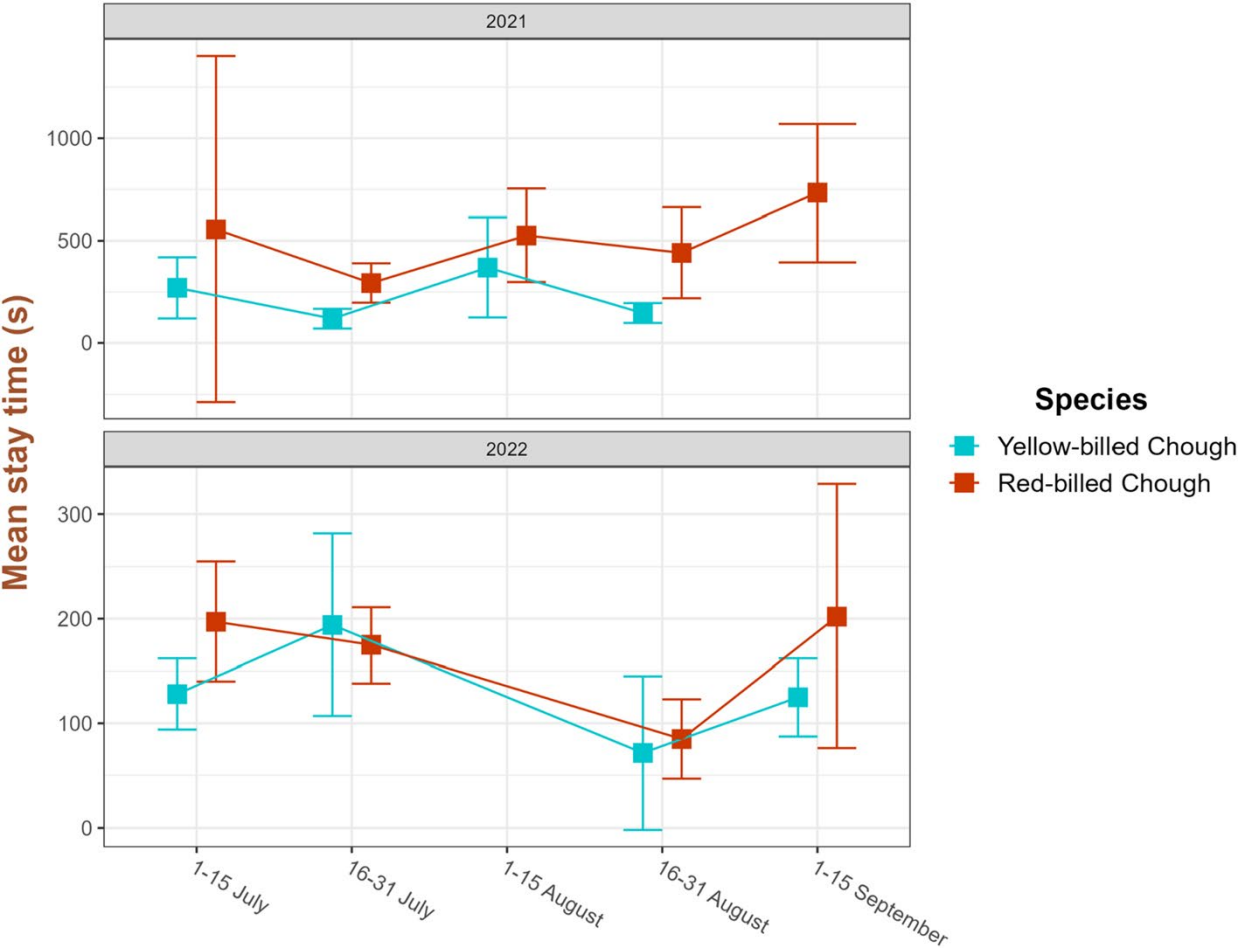
Seasonal trends in stay times for the two chough species in the years 2021 and 2022. Note that the scale of the ordinates is different in the 2 years: consequently, the differences between the two species are much wider in 2022 than in 2021.

For this reason, we considered the stay times for each species separately, and tested for an effect of year and flock size in monospecific flocks. The results showed stay times of both species to be significantly shorter in 2022: the decrease was greatest in the red-billed chough, by almost two-thirds (from an average of 473 s in 2021 to an average of 176 s in 2022, F_1,28_ = 18.04; *P* < 0.0002; Table S2); whereas a less conspicuous reduction was found for the yellow-billed chough (from 210 s in 2021 to 134 s in 2022, F_1,17_ = 4.31; *P* < 0.05; Table S3) (Fig. 2). It is worth mentioning that the difference in the stay times between the two species was most evident in 2021, with the red-billed chough staying on pastures on average two times longer than the yellow-billed chough in all the months of observations. Conversely, in 2022 the difference was considerably reduced, with monthly stay times being similar for the two species (Fig. 2).

By testing for the effect of flock type (monospecific vs mixed) in each year separately, we were able to detect a clear effect of mixed flocking on red-billed choughs in 2021. In that year, red-billed choughs stayed on pastures for an average of 473 s when in monospecific flocks (as indicated above), but only 146 s when in presence of the yellow-billed choughs (F_1,67_ = 9.45; *P* < 0.0031; Table S4). Notably, this difference was absent in 2022 (F_1,195_ = 1.22; *P* = 0.271; Table S5).

### Flock size

In both years, monospecific flocks of yellow-billed choughs were significantly more numerous than those of red-billed choughs. In 2021, we counted an average of 28.8 yellow-billed and 6.7 red-billed choughs per flock (significant difference, F_1,197_ = 33.97; *P* < 0.0001; Table S6), whereas in 2022 the numbers had dropped to 18.7 yellow-billed choughs and 8.0 red-billed choughs (F_1,210_ = 20.92; *P* < 0.0001; Table S7). Intraspecific variations between years were not significant (*P* > 0.300 for both species).

### Propensity to mixed flocking

**Figure 3:**
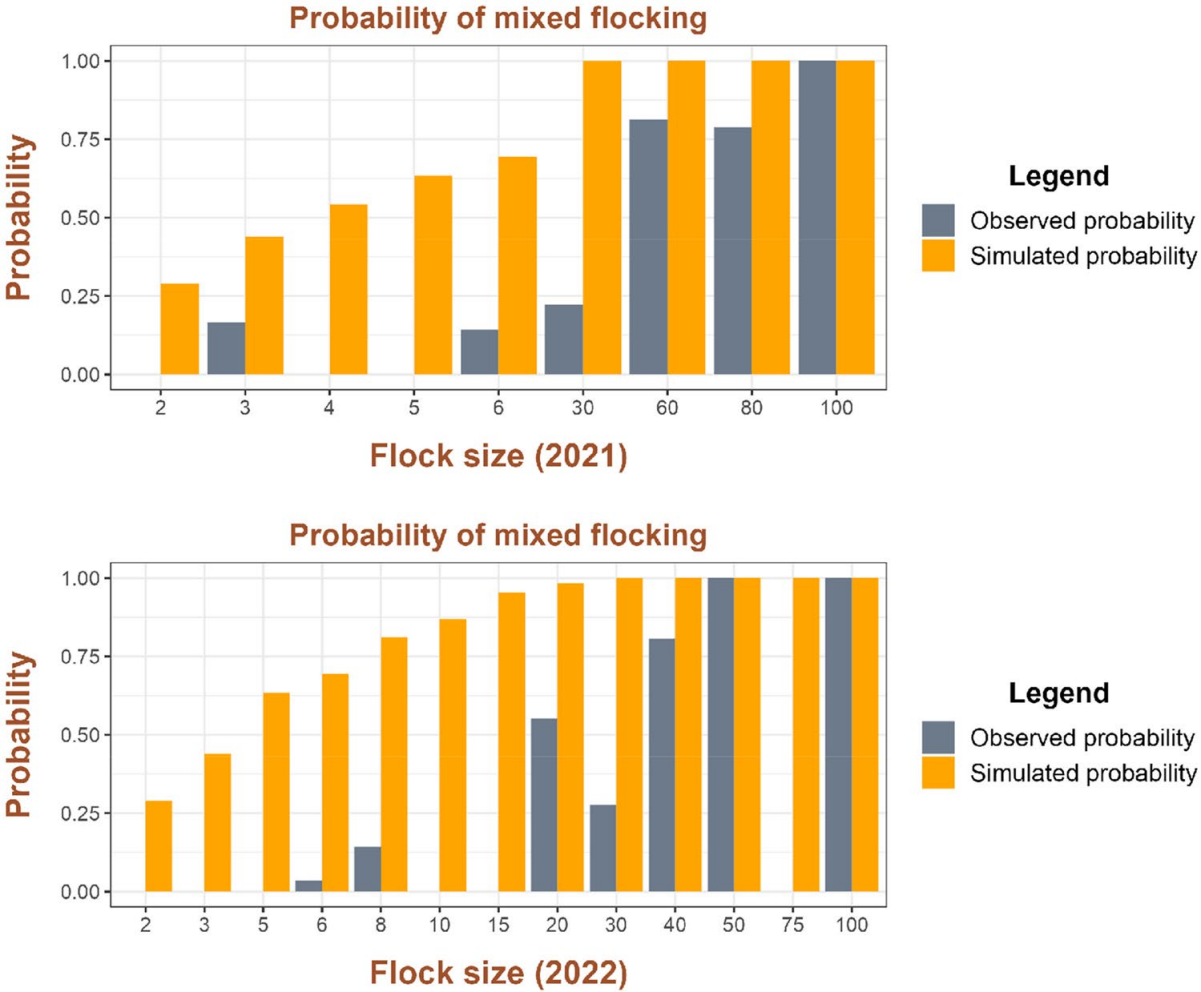
Propensity to mixed flocking in the years 2021 and 2022. The simulated probabilities based on random mixing are almost always significantly higher than the observed ones.

In both years, the propensity to mixed flocking was very low, especially when flocks were small. Indeed, simulated probabilities were always significantly higher than the observed probabilities, except for a flock size of 100 in 2021 and flock sizes 50 and 100 in 2022 (Fig. 3).

### Foraging home ranges

**Figure 4:**
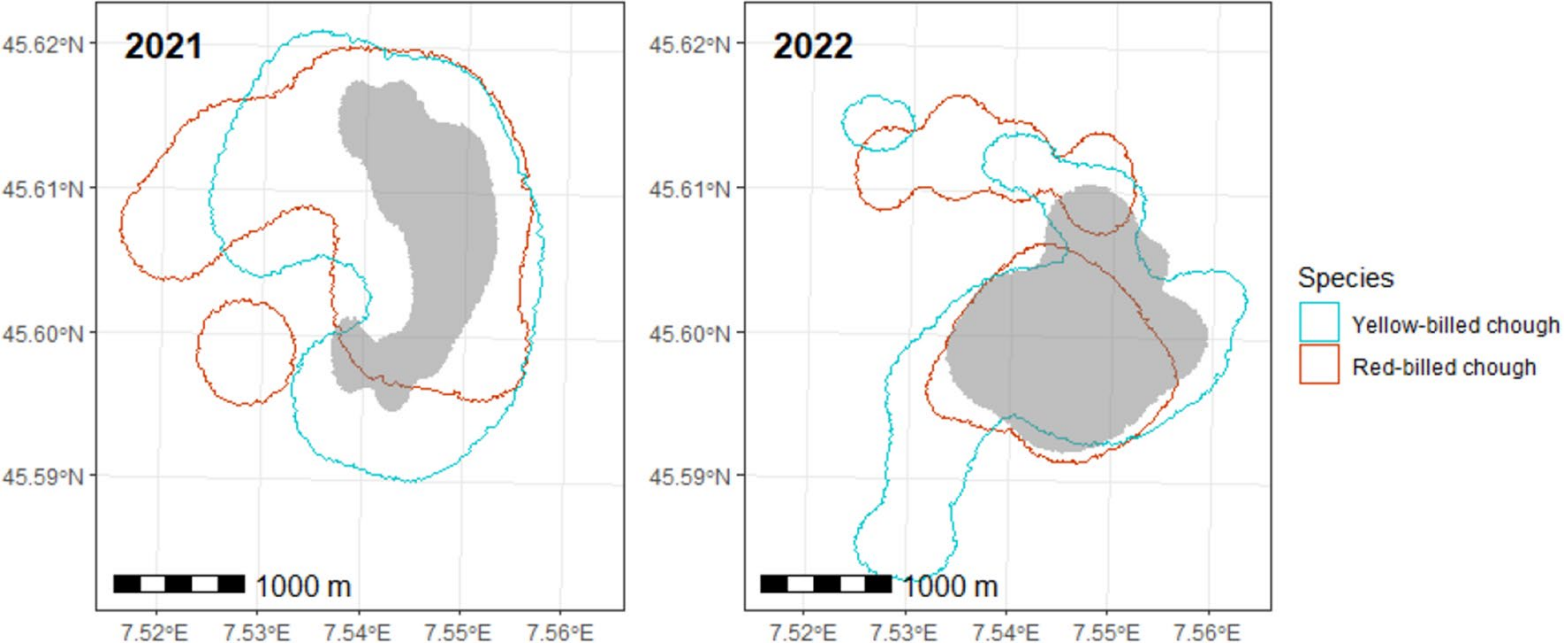
Home ranges of the foraging flocks in the years 2021 and 2022. The degrees of latitude and longitude are shown. Grey polygons represent the areas where both species were observed to forage together.

In both years, each species shared about the 56% and the 43% of their foraging home range with the other species in 2021 and 2022, respectively. The areas used by mixed flocks made up 24% and the 41% of home ranges of the two species in 2021 and 2022, respectively (Fig. 4).

### Altitudinal ranges

**Figure 5:**
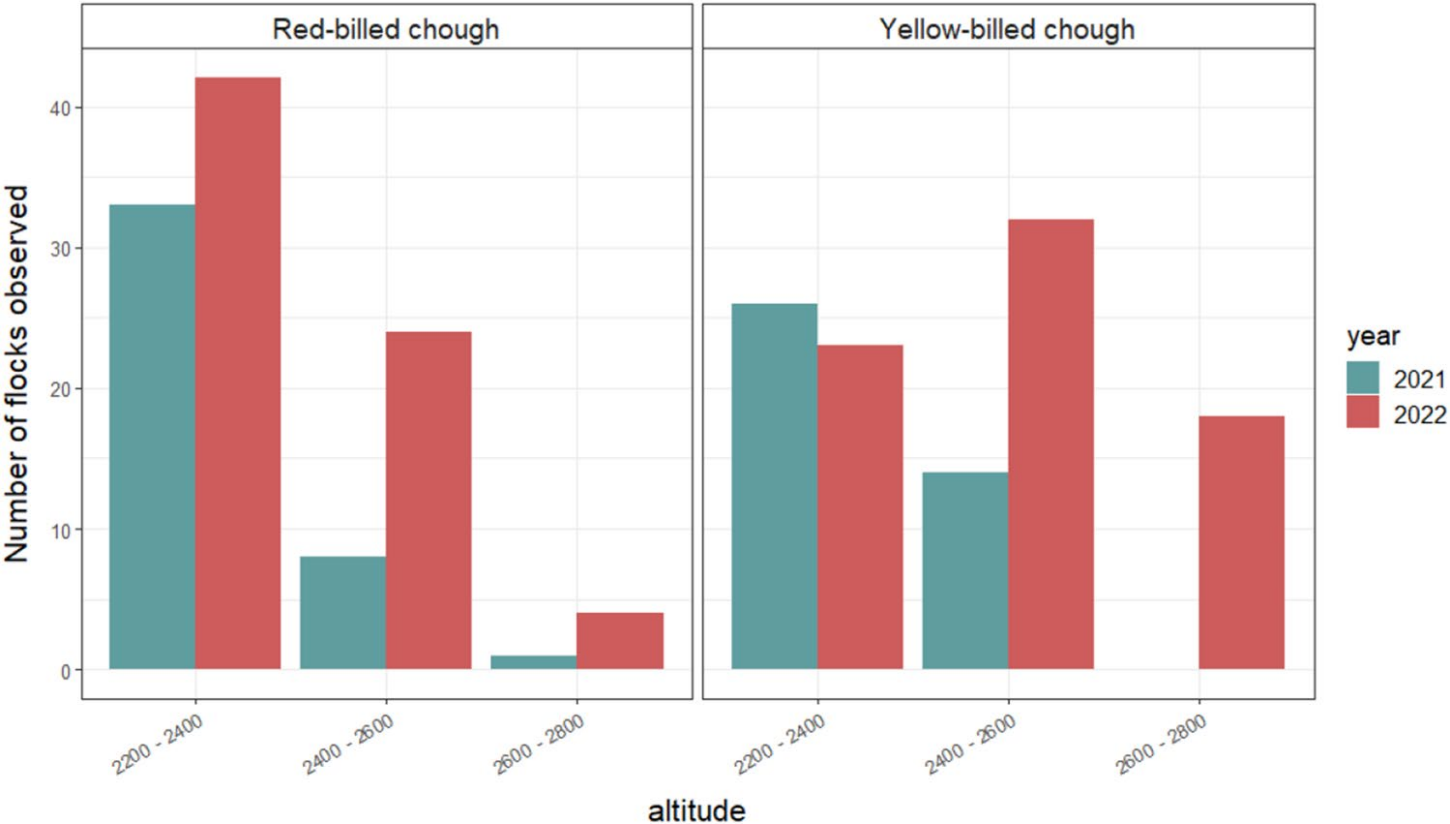
Altitude distribution of the foraging flocks for the years 2021 and 2022. Altitude intervals are in metres.

Birds foraged between 2200 and 2800 m a.s.l. The yellow-billed chough, but not the red-billed chough, significantly increased its altitudinal range in 2022, when many foraging flocks were observed at very high elevations, above 2600 m a.s.l. (*P* = 0.03, Fig. 5).

## Discussion

The present study took advantage of the peculiar climatic context to compare several traits of the foraging ecology of the yellow-billed chough and the red-billed chough between the years 2021 and 2022, namely foraging stay time, flock size, propensity to mixed flocking, foraging home range, and altitudinal range of the two species.

We detected no evident differences between the 2 years in relation to flock size, propensity to mixed flocking and foraging home ranges. Flocks of yellow-billed choughs were larger than those of red-billed choughs, in keeping with the relative abundances of the two species in many other valleys in the north-western Italian Alps (AR personal observations). We confirmed a low propensity to mixed flocking^38^, which was particularly low in the case of small flocks. We also showed that the area frequented by the mixed groups was much smaller than that frequented by monospecific flocks, hence suggesting that the low propensity to mixed flocking is also a matter of home range and spatial ecology.

Conversely, we detected significant differences in stay time and altitudinal range between the two years. The stay times for both species decreased significantly from 2021 to 2022 with regards to the foraging behaviours of individuals within monospecific flocks. The reduction was most dramatic for the red-billed chough (stay times decreased by almost two-thirds), but much less so for the yellow-billed chough. The differences in the stay times between species reflect differences in their foraging techniques. They are usually shorter in the yellow-billed chough because it relies on epigean food items, which are relatively easy to detect and catch on sight. Vice-versa, the stay times of the red-billed chough are longer because it relies on underground items, which cannot be detected by sight; animals are therefore forced into time-consuming activities such as probing and digging^37^. Insects constitute the main prey of both corvid species during the summer months^35^. The presence of epigeal insects can be assessed indirectly by passing a stick through the grass and counting the insects that are flushed out^24,26,33^. Vice-versa, to our knowledge, there is no practical method for assessing the presence of underground insects. In any case, the evaluation of food abundance has never been attempted and, accordingly, no data exist on how climatic variations between years may have affected the abundances of local food sources. That said, certain effects of temperature and humidity on insects are well known. Warm weather allows insect larvae to mature faster, whereas dry weather is more likely to trigger survival mode in adult insects (aestivation). Typically, soil microarthropod abundance is positively related to soil moisture content^39^. If we consider the hypogeal insects that the red-billed chough feeds on, their abundances may have diminished in 2022 with respect to 2021 as a direct consequence of drought or due to the warmer temperatures stimulating their emergence out of the ground. For example, it is known that an earlier snowmelt brings forward the emergence of soil-hibernating arthropods such as Coleoptera, Diptera, Hemiptera, Hymenoptera and Araneae^40^. The net result could have been a reduction in the red-billed chough's food availability. We can hypothesise, therefore, that the red-billed choughs were able to evaluate the scarcity of their food source through successive subterranean probing of the ground, leading them to abandon the foraging patch. The prolonged drought may also have hardened the soil, making the red-billed chough's probing and digging activities more difficult.

The two species influence each other when together in a mixed flock. Moreover, in these flocks, red-billed choughs are usually outnumbered by yellow-billed choughs (^38^, and in the present study). Consequently, the relative restlessness of the yellow-billed chough can significantly affect the congener, which is induced to interrupt its stays when yellow-billed choughs fly away^38^. This interspecific influence was confirmed in 2021, but not in 2022. This is most likely because the dramatic reduction in the duration of the red-billed chough’s stay times in 2022 would have led to the two species displaying, on many occasions, similar stay times. As a result, the yellow-billed chough no longer had the opportunity to induce any change to the congener’s behaviour, which had already been altered, most probably by climate-induced means.

Both species foraged between 2200 and 2800 m a.s.l., but the elevational distribution of the yellow-billed chough was significantly greater in 2022 compared with 2021 (many foraging flocks were observed above 2600 m asl), whereas that of the red-billed choughs was unchanged. The yellow-billed chough is particularly well adapted to high altitude. It has been reported as foraging on leftovers as high as 8000 m on Mount Everest^41^ and it has been found nesting as high as 6500 m^42^. Therefore, it is not surprising that under very hot conditions yellow-billed choughs will move higher, possibly looking for patches with better trophic availability (e.g., a greater availability of flying and oreophilic arthropods). Alternatively, this upward shift could be a response to the birds’ thermoregulation needs. Birds have no sweat glands, so the primary way of dissipating excessive heat is by panting and searching out shaded resting spots during the high sun. We did not collect data on thermoregulation behaviours, but during the summer of 2022 we repeatedly noticed both the red- and yellow-billed choughs with their beaks open or sheltering in the shade of large rocks. These observations call for future detailed study of the thermoregulation of upland birds^43,44^, even though they do not risk severe decline due to rising temperatures as predicted for arid zone birds^45,46^. In this framework, the movement of yellow-billed choughs towards higher elevations can be simply interpreted as their way of cooling off. The lack of an altitudinal rise in the red-billed chough is difficult to explain, but it could simply depend on the fact that this species suffers less from the heat given that it is not a true mountainous bird. In fact, in some parts of its range, it is also known to nest on sea cliffs.

Summing up, all the differences in the foraging behaviours observed between the two years could be explained as direct or indirect (via food availability and/or soil hardening) responses to the climatic extremes that characterised the year 2022. The fact that marked climatic alterations between subsequent years led to immediate significant changes in the foraging ethology and ecology may be surprising, however, this immediate response is in keeping with the extraordinary behavioural flexibility displayed by wildlife^47,48^. Indeed, many wild bird species quickly responded to the Covid-19 shutdown by changing their diet, song activity, daily routine, and habitat use^49–52^.

That said, even if the results are in line with the hypothesis that large climate variations may disrupt the foraging ecology of mountain birds, we must recognize that it is not possible to draw solid conclusions from just two years of observations. Therefore, further field research will be required to test the hypothesis with a more robust set of data.

## Material and methods

### Study area

The study area is the Dondena basin, located at the head of the upper Champorcher Valley (45°37′ N, 7°37′ E) in the northwestern Italian Alps. The entire study area is above the treeline, made up of typical alpine tundra habitats, namely flat pastures, steep grassy slopes, and rocky walls that culminate in multiple peaks, all of which exceed 3000 m in altitude (e.g., Mont Glacier 3185 m a.s.l., Rosa dei Banchi, 3164 m a.s.l.). Its grasslands are diversified and dominated, according to the very local edaphic conditions and exposures, by grasses such as *Sesleria caerulea*, *Elyna myosuroides*, *Carex curvula*, *Nardus stricta* and *Festuca luedii*^53^. In the spring these grasslands host blooms of many flower species, including *Arnica montana*, *Aster alpinus, Nigritella nigra*, *Polygonum bistorta*, *Pulsatilla alpina* and *Helianthemum nummularium*. Bush formations made up of *Juniper* spp., *Rhododendron ferrugineum*, *Vaccinium myrtillus* and *V. gaultherioides* are present in some areas.

### Field methods

Observations were carried out from June to September in 2021 and 2022, 9–12 days a month depending on weather conditions (Table S8). The presence of birds was detected from vantage points that allowed the whole basin to be viewed using binoculars (Zeiss 10 × 30) and spotting scopes (Bushnell Spacemaster 15–45 × 60) with tripods.

For the evaluation of foraging behaviour, we focused on the stay time; being the duration of time (measured with the use of a digital chronometer) a focal individual spent foraging on a pasture, starting the moment the individual landed on a patch of grass and ending upon take off.

Flocks were investigated by recording their composition (single species versus mixed flocks), size (i.e., the number of individuals making up the group) and localization (UTM coordinates and altitude, collected with the GPS devices Garmin eTrex 10 and eTrex 30). Both species exhibit very gregarious behaviour, and it is not unusual to observe choughs flying in large-scale flocks encompassing all local individuals. Therefore, we assumed that the largest flying flock observed (and photographed to count individuals) represented the actual total number of individuals foraging in pastures at that given moment (in keeping with^36^).

## Data analysis

### Climate

Climatic data (temperature, rainfall, and snowfall) were provided by the Functional Center of the Autonomous Region of Aosta valley (Civil Protection and Fire Brigade Department) using data collected from the local weather station. The effect of year, month, and their interaction on temperature was tested using generalised least squares (GLS) with an autoregressive–moving-average (ARMA) correlation structure for considering temporal autocorrelation. The ARMA correlation structure implied the Julian day nested within the year, and the autoregressive order and the moving average order were set to 2 and 0, respectively. The effect of year, month, and their interaction on precipitation (rainfall + snowfall) were tested using a hurdle model with a zero inflated gamma distribution with the logarithm as the link function for the conditional model. For both temperature and precipitation, the best model was identified by comparing competing models with a likelihood ratio test.

### Stay times

The effect of the predictor variables on stay times was tested using linear mixed effect models (LMMs) for both species considered jointly and separately. Correlated variables were removed prior to the analysis by looking at their Spearman correlation. Predictors with a correlation lower than 0.7 were kept for LMM analysis. After the removal of correlated variables, we used the stay time as the response variable for all models and flock size and year as predictors. Moreover, species identity was used as a predictor when analysing the two species jointly. Collection date was used as a random effect, and stay times were transformed by the natural logarithm to fulfil the LMM assumptions. The function *lmer* of the R^54^ package lmerTest^55^ was used to estimate the models and to calculate the *p*-values. *P*-values were calculated using Satterthwaite’s method for denominator degrees-of-freedom and F-statistic with the *ANOVA* function of the same package.

### Flock size

Flock size differences between 2021 and 2022 were tested by means of separate LMMs for each species, using year as the fixed effect and collection date as the random effect.

### Mixed flocking

To study mixed flocking, we compared the observed frequency with those expected by chance. The expected frequency was calculated using a random sampling approach. At first, we estimated the population size of each species as the maximum number of individuals found during the field activity. Secondly, we sampled *n* individuals without replacement from the pool of individuals of the two species, where *n* corresponds to each of the observed flock sizes found. This procedure was repeated 10,000 times. For each flock size, the expected frequency was then calculated as the number of times a mixed flock was achieved divided by 10,000. Observed and expected frequencies were compared with a binomial test using the function *binom.test* of the R package stats^54^. Results were reported for flock sizes occurring more than 10 times to avoid spurious results due to a small sample size.

### Foraging home ranges

The foraging home ranges were estimated using flock coordinates recorded in 2021 and 2022 for each species separately and for mixed flocks using the function *hypervolume* of the R package hypervolume^56^. Observations collected within the same year were aggregated to describe a unique foraging home range for each year. Specifically, hypervolumes were calculated using a Gaussian kernel density estimation method and the method “plug-in” as the kernel bandwidth estimator.

### Altitudinal ranges

Differences between the altitudinal foraging distributions of the two species for the two years were compared using Chi-square tests, the *P* values for which were computed using Monte Carlo methods.

### Supplementary Information

Supplementary Tables.

## Data Availability

Data are available from the first author upon reasonable request.
