# Supplementary material for: The foraging ecology of yellow-billed and red- billed choughs changed between two climatically different years
Source: Sci Rep. 2023 Nov 27;13:20908. doi: 10.1038/s41598-023-46336-0 (PMC10684611; doi:10.1038/s41598-023-46336-0)
Supplement: Supplementary file 1 — Supplementary Tables. [file 41598_2023_46336_MOESM1_ESM.pdf]

The foraging ecology of yellow-billed and red- billed choughs changed between two climatically different years

Antonio Rolando[1], Cecilia Basso[1], Nicolò Brunelli[1], Massimo Bocca[2], Alex Laini[1*]

[1]Department of Life Sciences and Systems Biology, Turin University, via Accademia Albertina 13, 10123 Turin, Italy

[2]Société de la Flore Valdôtaine, via J.-B. de Tillier 3, 11100 Aosta, Italy

*Corresponding author: Alex Laini

e-mail: alex.laini@unito.it

Table S1. Estimates of random effects and fixed effects for the mixed effect model with both species. Confidence intervals (2.5 and 97.5, obtained with the profile method) and p-values (Wald test) are also reported. Random effects were not tested for significance.

**variable**	**estimates**	**2.5**	**97.5**	**p_value**
day	0.38	0.27	0.54	NA
residual	0.94	0.89	1	NA
intercept	6.18	5.89	6.47	<0.001
red-billed chough	0.29	0.11	0.46	0.0014
flock size	-0.01	-0.01	0	0.0003
year_2022	-0.68	-1.02	-0.35	0.0002

Table S2. Estimates of random effects and fixed effects for the mixed effect model of the yellow-billed choughs. Confidence intervals (2.5 and 97.5, obtained with the profile method) and p-values (Wald test) are also reported. Random effects were not tested for significance.

**variable**	**estimates**	**2.5**	**97.5**	**p_value**
day	0.22	0	0.47	NA
residual	0.94	0.85	1.06	NA
Intercept	5.87	5.58	6.17	<0.0001
year_2022	-0.38	-0.76	0	0.0529

Table S3. Estimates of random effects and fixed effects for the mixed effect model of the red-billed choughs. Confidence intervals (2.5 and 97.5, obtained with the profile method) and p-values (Wald test) are also reported. Random effects were not tested for significance.

**variable**	**estimates**	**2.5**	**97.5**	**p_value**
day	0.38	0.24	0.59	NA
Residual	0.98	0.9	1.07	NA
Intercept	6.55	6.24	6.87	<0.001
year_2022	-0.86	-1.26	-0.45	0.0002

Table S4. Estimates of random effects and fixed effects for the mixed effect model of flock type in 2021. Confidence intervals (2.5 and 97.5, obtained with the profile method) and p-values (Wald test) are also reported. Random effects were not tested for significance.

**Variable**	**estimates**	**2.5**	**97.5**	**p_value**
Day	0.34	0	0.63	NA
Residual	1.09	0.96	1.25	NA
Intercept	5.61	5.05	6.17	<0.0001
Monospecific	0.94	0.34	1.53	0.0031

Table S5. Estimates of random effects and fixed effects for the mixed effect model of flock type in 2022. Confidence intervals (2.5 and 97.5, obtained with the profile method) and p-values (Wald test) are also reported. Random effects were not tested for significance.

Variable	estimates	2.5	97.5	p_value
Day	0.42	0.23	0.67	NA
Residual	0.88	0.79	0.97	NA
Intercept	5.95	5.51	6.39	<0.001
Monospecific	-0.23	-0.64	0.18	0.2714

Table S6. Estimates of random effects and fixed effects for the mixed effect model of flock size in 2021. Confidence intervals (2.5 and 97.5, obtained with the profile method) and p-values (Wald test) are also reported. Random effects were not tested for significance.

variable	estimates	2.5	97.5	p_value
Day	10.87	6.33	16.77	NA
residual	20.93	18.91	23.2	NA
intercept	26.15	19	33.13	<0.0001
red-billed chough	-19.65	-26.31	-13.07	<0.0001

Table S7. Estimates of random effects and fixed effects for the mixed effect model of flock size in 2022. Confidence intervals (2.5 and 97.5, obtained with the profile method) and p-values (Wald test) are also reported. Random effects were not tested for significance.

variable	estimates	2.5	97.5	p_value
Day	7.59	4.19	11.94	NA
residual	16.4	14.87	18.12	NA
intercept	18.71	13.76	23.7	<0.0001
red-billed chough	-10.87	-15.54	-6.21	<0.0001

Table S8. Sampling date in 2021 and 2022.

Date	2021	2022
July	14, 27, 28, 29,	5, 6, 7, 11, 12, 13, 22, 23, 25, 26, 27
August	2, 3, 12, 13, 14, 23, 24, 25, 26	21, 22, 23
September	7, 8, 9	5, 6, 7
